# Fabrication of Monodisperse Flower-Like Coordination Polymers (CP) Microparticles by Spray Technique

**DOI:** 10.3390/nano7090237

**Published:** 2017-08-25

**Authors:** Wen-Ze Li, Yuan Zhou, Fuchun Liu, Yunong Li, Ming-Jian Xia, En-Hou Han, Tieqiang Wang, Xuemin Zhang, Yu Fu

**Affiliations:** 1 Department of Applied Chemistry, Shenyang University of Chemical Technology, Shenyang 110142, China; 2 College of Sciences, Northeastern University, Shenyang 110819, China; m13234040986@163.com (Y.Z.); liyunong@mail.neu.edu.cn (Y.L.); xiamingjian0927@163.com (M.-J.X.); caswtq@163.com (T.W.); zhangxuemin@mail.neu.edu.cn (X.Z.); 3 Key Laboratory of Nuclear Materials and Safety Assessment, Institute of Metal Research, Chinese Academy of Sciences, Shenyang 110016, China; fcliu@imr.ac.cn (F.L.); ehhan@imr.ac.cn (E.-H.H.)

**Keywords:** coordination polymers, metal-organic frameworks (MOF), spray, flower-like microparticles

## Abstract

In this manuscript, we have developed an efficient spraying method to successfully fabricate a series of flower-like coordination polymers (CP) microparticles, including Co/BDC (1,4-benzenedicarboxylate) metal organic frameworks (MOF) and infinite coordination polymers (ICP) microparticles, as well as Ni-Co/BDC MOF and Zn/DOBDC (2,5-dioxido-1,4-benzenedicarboxylate) MOF. The spraying method has shown high efficiency and universality in synthesizing the flower-like CP. The crystalline structure can be adjusted by varying the solvent composition in the spraying process. SEM observation demonstrated the MOF and ICP microparticles possess the similar flower-like structure, which is composed of nanoflakes with smooth surface, and the flower-like microparticles could be monodisperse with as low as 5% polydispersity. Moreover, the fabrication of the flower-like CP microparticles by spraying has a wide operation window, because there is no need to precisely control the experiment conditions, like solvents, concentration, and spray order. Due to the practicality of spray technique, this work would pave the way for the manufacture of the flower-like materials and have great potential in applications of catalysis, sensor, energy storage, and so on.

## 1. Introduction

Coordination polymers (CP) are a class of organic-inorganic hybrid materials, which are based on metal ions linked by polydentate organic ligands [1,2,3,4]. The CP family involves two important members: metal-organic frameworks (MOF) and infinite coordination polymers (ICP). Normally, CP with highly crystalline structures and a well-defined framework are referred to as MOF [5], and the ones with amorphous structures or without definite crystalline structures are referred to as ICP [6]. MOF which possess inherent porosities, high surface, and uniform channels have emerged as an intriguing class of porous materials with a high potential for applications in separation, storage, and catalysis [7,8,9,10]. ICP have the advantage over MOF in terms of synthesis. Typically, MOF require more effort to choose the appropriate synthesis conditions to obtain a pure crystalline product, such as solvent, temperature, and so on [11]. However, the synthesis of ICP is simpler, which presents ICP as having more customization in their structure, composition, and morphology. This makes ICP attractive for many applications where conventional MOF are not suitable or ideal given their static morphology, such as biologically-relevant imaging, labelling, drug delivery, and so on [6]. Due to their respective properties and the functionality hosted by both organic and inorganic building blocks, MOF and ICP have attracted tremendous attention during the past decade.

Among the studies on CP, considerable efforts have been devoted to the exploration of their nano- and microstructures, namely the crystal size, morphology, and the shape of crystal aggregates [12,13,14,15]. The desired structures could present new opportunities for applications and lead to the discovery of new intrinsic phenomena [16,17,18]. In addition, the CP with suitable structures for device manufacture could bridge the gap between fundamental CP science and prospective applications [19]. Almost all CP synthesis methods have been employed to prepare nano- and microstructures, such as direct precipitation, hydrothermal reaction, microwave/ultrasound driven synthesis, electrochemistry, and so on [16,20,21,22]. Hence, a variety of nano-and microstructures have been achieved, including spheres, cubes, rods, wires, sheets, ribbons, straw-sheaf, and so on [23,24,25,26,27,28,29]. However, structuring CP at the nano- and micro-scales is a nascent field and it is still a challenge to fabricate complex or desired CP nano- and microstructures through a facile and controllable approach.

**Scheme 1:**
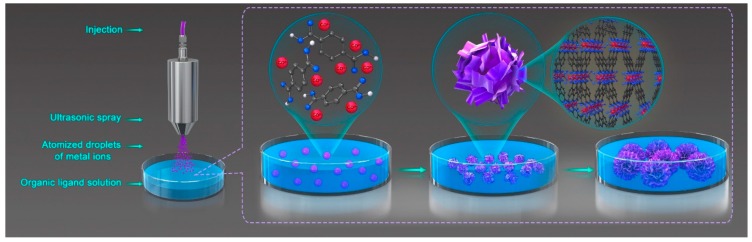
The fabricationprocess of flower-like microparticles.

The flower-like microparticles with nanoflakes are an intriguing class of nano- and microstructures. The flower-like structures could be considered as an aggregation of two-dimensional nanosheets, where the surface of the nanosheets is fully exposed to the environments and facile to be accessed. Therefore, the flower-like microparticles are promising candidates for applications in catalysis and analytical science [30,31,32,33,34]. However, the current flower-like structures are mainly made of metal and metal oxide, but the ones made of CP components are rarely reported so far [35]. In this work, we developed a facile method to fabricate uniform flower-like CP particles with nanoflakes by spray technique. As shown in Scheme 1, the metal ion solution was nebulized by ultrasonic sounds and then sprayed on the surface of the organic ligands solution. The gentle touch of the droplet and the solution gave rise to a steady interface, where the crystal inclined to grow in the lateral direction and therefore generated the CP nanosheets [27]. Consequently, the resulting nanosheets concentrated in the zone of the droplets, making it possible for the nanosheets to aggregate further. Under appropriate experimental conditions, the nanosheets would be assembled crosswise into the flower-like structures. We employed Co^2+^ and 1,4-benzenedicarboxylate acid (H_2_BDC) as building blocks to fabricate CP flower-like structures by spray technology. Through adjusting the solvents, MOF and ICP flower-like microparticles with nanoflakes were successfully achieved. The polydispersity of the microparticles was around 5%, close to monodisperse. To the best of our knowledge, such monodisperse microflowers are barely reported. In addition, to demonstrate the universality of the method, the flower-like microstructures of Ni-Co/BDC MOF and Zn/DOBDC MOF were also prepared by spraying the corresponding nebulized metal ion solutions into the ligands solutions. As is well-known, the spray is an easy-handle technique and has been widely used in industry [36]. Therefore, our investigation indicated that the utilization of spray is an efficient and industrialized means to fabricate CP flower-like microparticles, paving a practical route to next-step applications.

## 2. Experimental Section

### 2.1. Materials

Cobalt acetate tetrahydrate [Co(CH_3_COO)_2_·4H_2_O], nickel acetate tetrahydrate, magnesium nitrate hexahydrate, 1,4-benzenedicarboxylate acid (H_2_BDC), [C_4_DABCO]Br and 2,5-dioxido-1,4-benzenedicarboxylate (DOBDC) were purchased from Energy Chemical (Shanghai, China). Dimethylformamide (DMF), phenyl epoxide, acetonitrile (CH_3_CN), and ethanol (C_2_H_5_OH) were purchased from Alfa Aesar (Beijing, China). All the reagents and solvents were used directly as supplied commercially without further purification.

### 2.2. Instruments

The ultraphonic spray nozzle and system was manufactured by Siansonic Technology Ltd (Beijing, China). The model of the ultrasonic generator is DP30 and the nozzle is ZPQ-S-95. The morphology of the as-prepared samples and the energy dispersive spectroscopy (EDS) spectroscopy were obtained by using a JEOL JSM-6700F field emission scanning electron microscope (SEM, JEOL Ltd., Tokyo, Japan). X-ray diffraction (XRD) data were collected on a Bruker D8 Advance instrument (Bruker Instrument Co. Ltd., Karlsruhe, Germany) in the 2θ range of 5°–50° using Cu K*α1* radiation (k = 1.54056 Å) at room temperature. Fourier transform infrared spectra (FT-IR) were recorded using a Bruker Tensor 27 FT-IR spectrometer (Bruker Instrument Co. Ltd., Karlsruhe, Germany) with KBr pellets. The surface area of sample was calculated by analyzing their nitrogen gas adsorption/desorption isotherms using the Brunauer–Emmett–Teller (BET) method (ASAP 2020M, Micromeritics Instrument Corp., Norcross, GA, USA). Thermogravimetric analysis (TGA) was performed on a TA Instrument 931 (Ta Instrument, New Castle, DE, USA). 

### 2.3. Preparation of Co/BDC CP Microparticles by Spray

The Co/BDC CP microparticles were fabricated by spraying cobalt acetate tetrahydrate solutions onto H_2_BDC solutions with the ultraphonic spray nozzle and system at room temperature (a picture of the equipment is shown in Figure S1).In the synthetic procedure of Co/BDC MOF microparticles, Co(CH_3_COO)_2_·4H_2_O, and H_2_BDC were respectively dissolved in a mixed solvent, which formed a ternary mixture of DMF/ethanol/water (*V*:*V*:*V* = 16:1:1), and the optimal concentrations of cobalt acetate tetrahydrate and H_2_BDC are both 0.021 mol·L^−1^. The Co/BDC MOF microparticles were produced by spraying 2 mL cobalt acetatetetrahydrate solution onto the 4.5 mL H_2_BDC solution in a glass culture dish at the flow rate of 36 μL/min. As for Co/BDC ICP microparticles, DMF was chosen as the solvent and the optimal concentrations of cobalt acetate tetrahydrate and H_2_BDC are both 0.21 mol·L^−1^. The Co/BDC ICP microparticles were produced by spraying 0.5 mL cobalt acetate tetrahydrate solution onto the 4.5 mL H_2_BDC solution in a glass culture dish at the flow rate of 36 μL/min. Once the nebulized solution of cobalt acetate tetrahydrate touched the surface of the H_2_BDC solution, the pink precipitates instantly occurred, which were directly collected by a glass substrate. After being washed with DMF and dichloromethane, the obtained sample was stored for further characterization.

Elemental analysis was used to ensure the molecular formula of Co/BDC MOF [7]. The empirical formula for Co(1,4-BDC) (DMF) is C_11_H_11_NO_5_Co (Molecular weight 296.10 g·mol^−1^), which translates into wt-% of C 44.61, H 3.74, N 4.73,Co 19.90. In accordance, the experimental wt-% were C 44.58, H 3.76, N 4.76, Co 19.88.

### 2.4. Catalytic Test

The flower-like microparticles of Ni-Co/BDC and Co/BDC as catalysts were tested using the reaction of phenyl epoxide and CO_2_ to produce phenyl cyclic carbonates. For a typical catalyst preparation, a precalculated amount of Co/BDC (22.3 mg), [C_4_DABCO]Br (18.8 mg), and phenyl epoxide (240 mg) were added into a 25 mL reaction flask. Subsequently, the reactants were continued stirring and kept at 100 °C for 30 h under mild conditions of atmospheric CO_2_ pressure (1 atm of pure CO_2_). The resulting product was purified by column chromatography for further testing.

### 2.5. BET and TGA Tests

To obtain the pure sample for BET and TGA tests. The pink precipitates were collected by centrifugation and washed several times with DMF and dichloromethane to remove the unreacted reactants. Then the product was further purified by Soxhlet extraction at 100 °C with methanol as solvent. Finally the dry sample was activated in an oven at 130 °C for 12 h.The obtained sample was stored for further tests.

## 3. Result and Discussion

The Co/BDC CP microparticles were fabricated by spraying the nebulized solution of cobalt acetate tetrahydrate onto the H_2_BDC solution as shown in Scheme 1. When the droplets of cobalt acetate tetrahydrate solution touched the surface of the H_2_BDC solution, visible pink precipitates instantly appeared, implying simultaneous formation of microparticles. To obtain different crystalline structures, two different solvents of ternary mixture (DMF/ethanol/water) and pure DMF were used, respectively.

**Figure 1:**
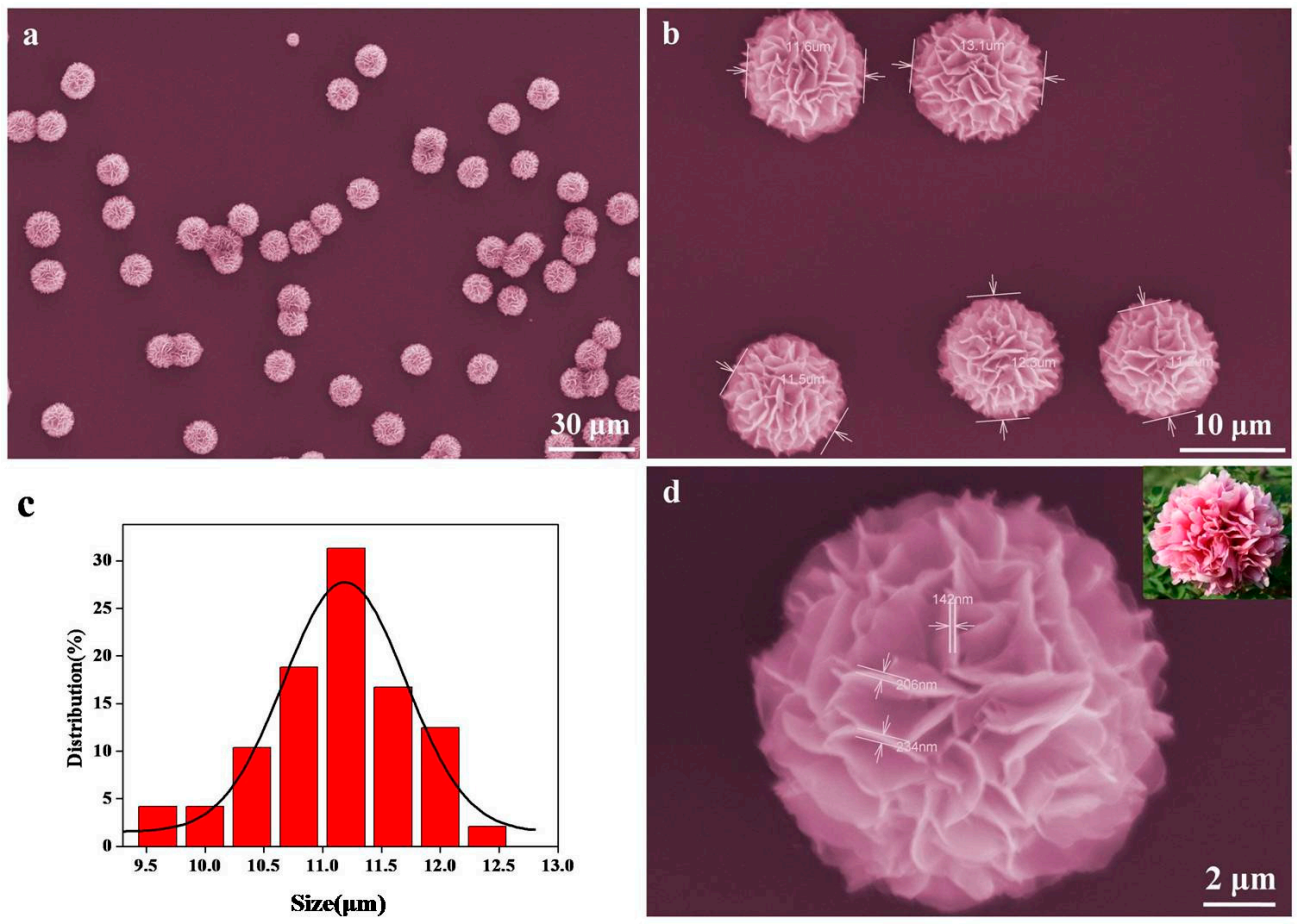
SEM images (**a**,**b**,**d**) and diameter distribution (**c**) of Co/BDC CP microparticles fabricated in the solvent of the ternary mixture. The insert in (**d**) is a natural peony.

**Figure 2:**
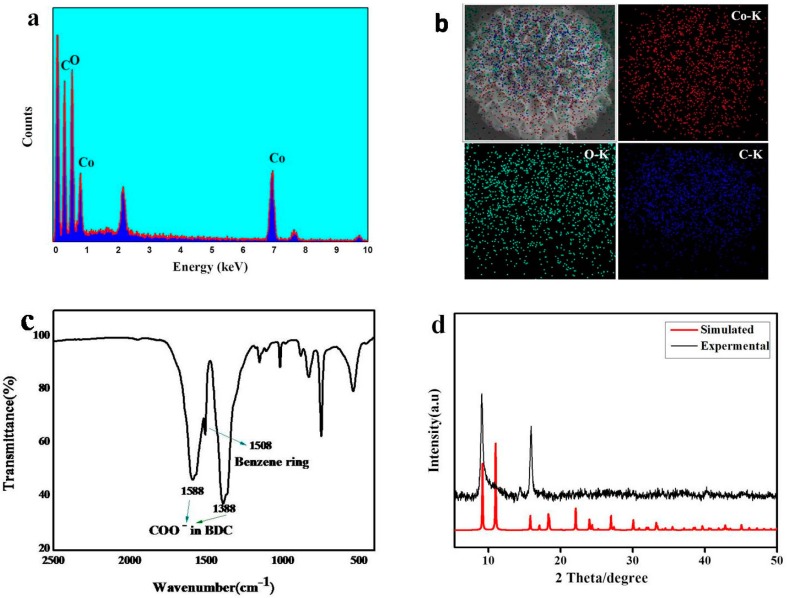
EDS spectrum (**a**); elemental mapping image (**b**); FT-IR spectrum(**c**) and XRD (**d**) of Co/BDC CP microparticles fabricated in the solvent of the ternary mixture. The structure of the simulated 3D reference was shown in Figure S3.

The microparticles prepared in the ternary mixture were firstly characterized by SEM. The SEM image (Figure 1a) showed that the uniform flower-like microspheres were separately dispersed on the surface. The diameter of the microspheres could be measured from the SEM image (Figure 1b). The diameter distribution was determined by summarizing the sizes of 50 individual particles as shown in Figure 1c, given that the microparticles diameters mainly ranged from 9.7 to 12.8 µm. Meanwhile, the statistical average value can be inferred to be 11.2 µm and the mean square can be 0.6 µm, suggesting that the relative standard deviation is only 5.0% and the flower-like microparticles are monodisperse. Figure 1d displayed a high magnification image of the microsphere. It was clearly shown that the microparticle was composed of nanoflakes with a smooth surface. These nanoflakes were interlaced together, forming a flower shape, just like a bloomed peony. According to the SEM images, the nanoflakes had sizes of several micrometers and had a thickness of about 200 nanometers, fitting the characteristic of two-dimensional materials. Furthermore, the composition of the flower-like microparticles was investigated by EDS, elemental mapping and FT-IR. The EDS graph manifested that the elements of Co, C, and O were incorporated into the microparticles (Figure 2a). The elemental mapping further demonstrated that the elements of Co, C, and O distributed uniformly in the microparticles (Figure 2b), indicating their homogeneity in the composition. In the FT-IR spectrum of the microparticles (Figure 2c), the characteristic peaks at 1508 cm^−1^ referred to a ring vibration in BDC. The bands of carboxylate groups were shown at 1588 cm^−1^ for antisymmetric stretching and 1388 cm^−1^ for symmetric stretching. The disappearance of the band of the nonionized carboxylate groups around 1684–1714 cm^−1^ revealed the H_2_BDC had been deprotonated and coordinated with Co^2+^ ions successfully. The crystalline structure of the microparticles was determined by XRD. The XRD pattern showed only two sharp and strong peaks of 9.1° and 15.9° (2θ) (Figure 2d), which could be indexed as the (-200) and (-011) crystallographic planes of MOF-71 crystal. The absence of the other peaks in the simulated pattern suggested that the microparticles were supposed to have a 2D MOF crystalline structure, confirming the observation in SEM. The above XRD characterization indicated that the Co/BDC microparticles fabricated by spray in the ternary mixture was a largely 2D layered material that is related to MOF structure. In addition, the obtained Co/BDC MOF was measured by BET and TGA. As showed in Figure S2, Co/BDC MOF showed a typical type IV isotherm with a hysteresis loop. The specific surface area of Co/BDC was calculated to be 170.1 m^2^·g^−1^ and the pore-size distribution was from 3 to 200 nm, suggesting the coexistence of structural pores as well as the pores between the nanosheets. TGA was measured to investigate the thermal stability of Co/BDC MOF (data not shown). The weight loss was observed from about 405 °C and terminated at approximately 700 °C, due to the decomposition of the MOF structure.

**Figure 3:**
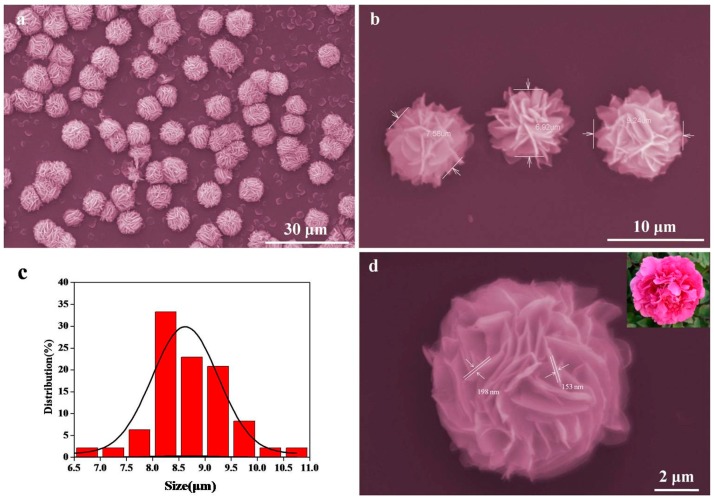
SEM images (**a**,**b**,**d**) and diameter distribution (**c**) of Co/BDC CP microparticles fabricated in the solvent of the pure DMF. The insert in (**d**) is a natural peony.

**Figure 4:**
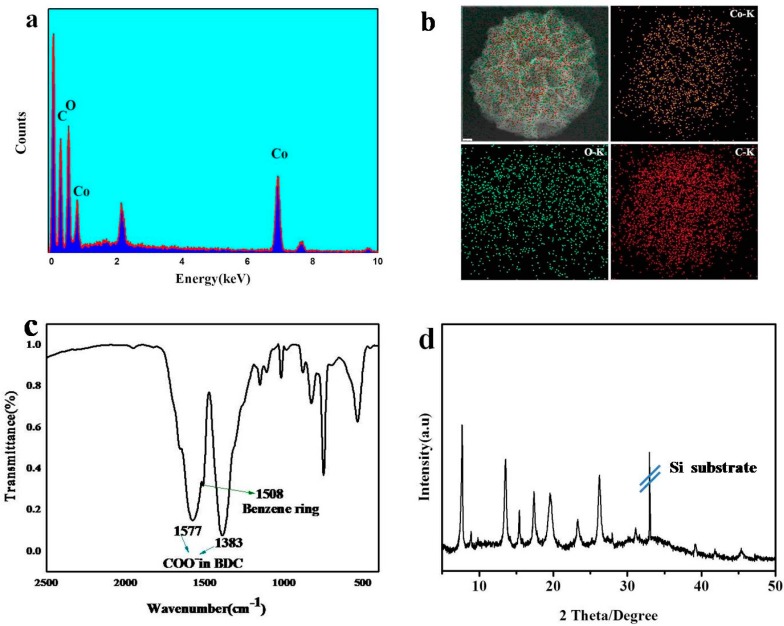
EDS spectrum (**a**); elemental mapping image (**b**); FT-IR spectrum (**c**) and XRD (**d**) of Co/BDC CP microparticles fabricated in the solvent of the pure DMF.

The other Co/BDC CP microparticles were obtained in pure DMF. The SEM image showed that the uniform flower-like microspheres were separately dispersed on the surface (Figure 3a). The diameter of the microspheres was measured (Figure 3b) and the diameter distribution was summarized by counting 50 individual particles as shown in Figure 3c. The diameter of the microparticles mainly ranged from 6.5 to 10.6 µm while the statistical average value was 8.7 µm and the mean square was 0.7 µm, suggesting the relative standard deviation was only 8.0%, close to monodisperse. Figure 3d displayed a high magnification image, which indicated that the nano- and microstruture of this microparticle was almost the same as the one of the Co/BDC MOF microparticle fabricated in the mixture solvent. The chemical composition of the microparticles was investigated by EDS, elemental mapping, and FT-IR as well. EDS graph (Figure 4a) demonstrated the elements of Co, C, and O were incorporated. The elemental mapping (Figure 4b) verified that the elements Co, C, and O distributed uniformly in the microparticles, revealing a homogenous composition in the sample. The FT-IR spectrum of the microparticles fabricated in DMF was identical to that of the MOF counterpart as shown in Figure 4c, indicating that the Co^2+^ ions have coordinated with the H_2_BDC ligands successfully. However, the XRD pattern of the microparticles (Figure 4d) was totally different from that of the MOF counterpart and even did not fit any standard of the known MOF [37]. The corresponding single crystal could not be obtained, which limited research on its accurate structure. Since the crystalline structure was not definite, the flower-like microparticles prepared in DMF were referred to as Co/BDC ICP temporarily.

**Figure 5:**
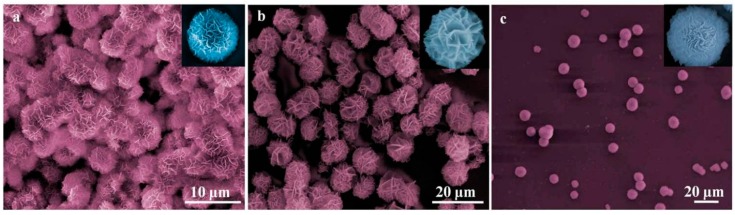
SEM images of (**a**) Co/BDC MOF fabricated with the solution concentration is 0.21 mol·L^−1^; (**b**) Co/BDC ICP fabricated in the solution concentration is 0.104 mol·L^−1^; (**c**) Co/BDC ICP sprayed in the reverse order. The inserts are the individual microparticles respectively.

The preparation of the flower-like CP microparticles through spray technique has a wide operation window. The above fabrication of MOF and ICP microparticles has suggested that, although the different solvents could result in different crystalline structures, the solvent made no difference on the flower-like morphology. Besides the solvent, the concentration, spray order, and flow rate do not have considerable influence on the formation of the flower-like structures, either. For instance, we have attempted to increase the concentration of starting materials by order of magnitude to fabricate Co/DBC MOF and decrease the concentration by one order magnitude to fabricate Co/BDC ICP. In both cases, the uniform flower-like microparticles were achieved as shown in Figure 5a,b. In addition, reversing the spray order—i.e., spraying the organic ligands solution into the metal ion solution—has been tried, which also produced flower-like structures as shown in Figure 5c.

**Figure 6:**
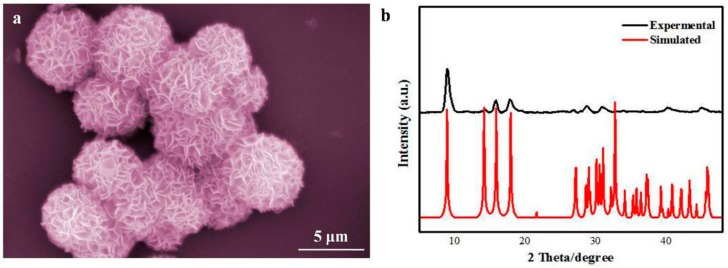
SEM images (**a**) and XRD (**b**) of Ni-Co/BDC MOF microparticles fabricated in the solvent of the ternary mixture. The structure of the simulated 3D reference was shown in Figure S4.

**Figure 7:**
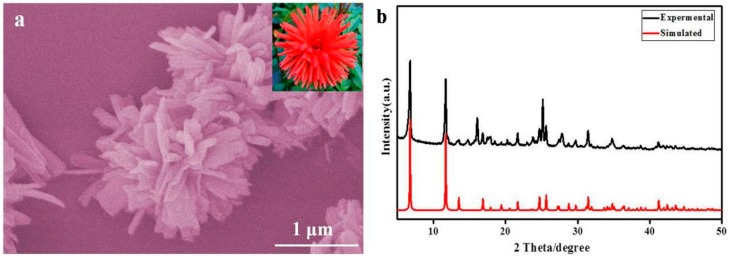
SEM images (**a**) and XRD (**b**) of Zn/DOBDC MOF microparticles fabricated in the solvent of the ternary mixture. The structure of the simulated 3D reference was shown in Figure S5. The insert in (**a**) is a natural chrysanthemum.

Besides the Co/BDC system, Ni-Co/BDC bi-metal CP and Zn/DOBDC CP were fabricated by spraying the Co^2+^ ion solution into the ligands solution as well. In the SEM images, Figure 6a showed the Ni-Co/BDC CP had the same morphology as Co/BDC CP and Figure 7a exhibited that the morphology of the Zn/DOBDC CP was different, but still presented a flower shape of chrysanthemum. The Ni-Co/BDC and Zn/DOBDC CP were also characterized by XRD. As shown in Figure 6b and Figure 7b, the XRD patterns were coincident with their simulated MOF XRD patterns respectively, confirming their accurate MOF crystalline structure and further exhibiting the universality of the spraying method.

**Figure 8:**
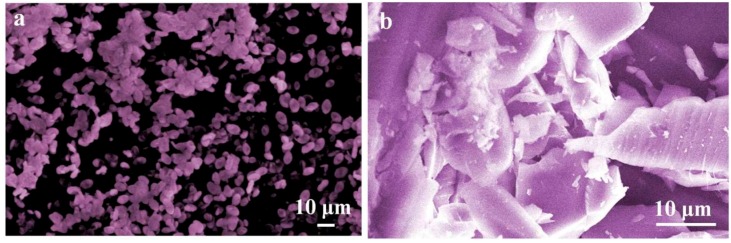
SEM images of (**a**) Co/BDC MOF; (**b**) Co/BDC ICP fabricated by dropping method.

In our further in-depth experiments, the spray technique can be proven to play a key role in the formation of the flower-like structure. When the solutions of metal ion and organic ligands were mixed by other means, such as pouring or dropping, the corresponding CP materials could be obtained, whereas the flower-like structures disappeared. For example, the dropwise addition of the Co^2+^ions into the ternary solvent solution of H_2_BDC and the DMF solution of H_2_BDC produced granule MOF particles (Figure 8a) and irregular ICP bulks (Figure 8b), respectively. The fabrication of such flower-like structures could be separated into two stages: generation of the nanosheets petals and crossing assembly of the resulting petals into a flower. Directly mixing the solutions of metal ion and organic ligands could not prepare the nanosheets, not to mention the following assembly. However, spray technique could match the fabrication conditions. As shown in Scheme 1, initially, due to the gentle touch, spraying the nebulized metal ion solution onto the surface of the organic ligand solution could give rise to a steady interface. At the interface, the CP was inclined to grow in the lateral direction, which led to the formation of the nanosheets. Subsequently, the resulted nanosheets aggregated in the zone of the droplets due to its low diffusion rate, which induced the assembly of the nanosheets’ “petals” into the individual “flower”. Although some fundamental issues including the details of the assembly process and the exact driving force cannot be clearly interpreted yet in this work, the spray technique with the practicality and efficiency has made itself a promising candidate for building up flower-like structures.

The anion in the metal salt is another essential factor for the formation of the flower-like microstructures. The nitrate and sulphate have been tried, but no flower-like microstructures were observed. For nitrate and sulphate, the precipitates did not occur instantly after spraying. It would take several hours for the products to precipitate. This suggested the reaction of acetate is much faster than that of nitrate and sulphate, which is likely because the acetate acid is a weak acid. The reaction speed is very important for the formation of the microstructures. After the solution of metal salt was sprayed on the surface of the organic ligand solution, the metal salt solution began to diffuse into the ligand solution. If the reaction is not fast enough, the two solutions will be mixed up, just like two solutions mixed by agitation, which cannot produce the desired microstructures. When the reaction is fast enough, just like that in our work where the product precipitates immediately after spraying, the product was generated with the diffusion of one solution into the other, which would meet the condition of fabricating the flower-like microstructures as discussed in the introduction.

**Scheme 2:**
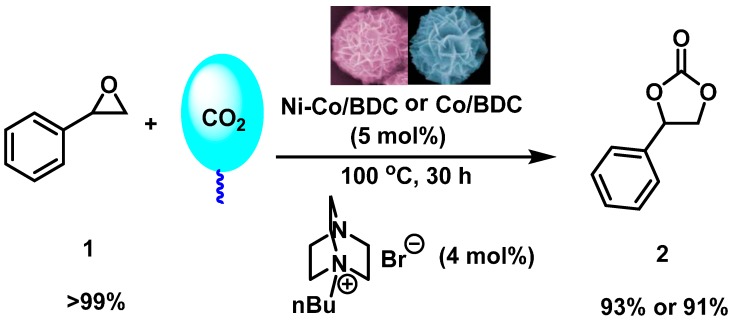
Flower-like microparticles of Ni-Co/BDC and Co/BDC catalyzed cycloaddition of CO_2_ with epoxides.

Furthermore, because of the multiple Lewis acid centers, full of nanosheets’ “petals”, and flower-like structures, these CP microparticles dramatically have an advantage in serving as a catalyst. Exactly, the flower-like microparticles have been found to exhibit excellent catalytic activity for the cycloaddition of CO_2_ and epoxide, which is an important organic reaction for CO_2_ conversion. Particularly, employing CP of Ni-Co/BDC and Co/BDC as the catalysts, the phenyl epoxide 1 can be completely converted into phenyl cyclic carbonates 2 in the high yields of 93 and 91%, respectively, under mild conditions of atmospheric CO_2_ pressure at 100 °C (Scheme 2). The probable reason may be that Lewis acid centers of Ni^2+^ and Co^2+^ could both activate CO_2_ and the oxygen in the epoxide, and the nanosheets and flower-like structures could increase the specific surface area of catalysts, thus both facilitating the cycloaddition to produce the valuable five-membered cyclic carbonates efficiently. This process is of great significance, turning waste gas CO_2_ to value-added compounds, which represents a promising field in view of sustainable development. In the previous work on this cycloaddition, the catalysts usually had the disadvantages of hard separation, low yields, high CO_2_ pressure (~5 MPa), and high reaction temperature (>100°C). Compared with the current catalysts—such as salen-type, ionic liquid-type, or other polymer salts—our catalyst is efficient and easy to handle with the simple synthesis process. Hence, with the aid of our catalyst, this cycloaddition could be successfully realized under mild conditions of atmospheric CO_2_ pressure and a relatively low temperature of 100 °C with high carbonate yield.

## 4. Conclusions

In summary, the spray technique has been utilized to prepare CP microparticles. Four kinds of flower-like CP microparticles—including Co/BDC MOF, Co/BDC ICP, Ni-Co/BDC MOF and Zn/DOBDC MOF—have been successfully fabricated by spraying the nebulized solution of metal ion into the organic ligand solution. The spraying method has a wide operation window. Using different solvents, concentrations, and spraying orders, the similar flower-like structures could be obtained. Moreover, the crystalline structure can be controlled by adjusting the solvents. It is worth mentioning that via spraying method, monodisperse or nearly monodisperse flower-like microparticles could be achieved, which are rarely produced by other methods. Such microparticles that would be promising building blocks in surface assembly also have great potentials in surface pattern and related applications. The work with regard to the assembly of monodisperse flower-like microparticles at the air-liquid interface is underway in our group.

## Supplementary Materials

The following are available online at http://www.mdpi.com/2079-4991/7/9/237/s1, Figure S1: The picture of the fabrication equipment, Figure S2: N_2_ adsorption–desorption isotherms of the Co/BDC MOF, Figure S3: The crystal structure of Co/BDC MOF [S1]. Color scheme for chemical representation: blue for Co, red for O, black for C, Figure S4: The crystal structure of Ni-Co/BDC MOF [S2], with the purple octahedral representing the Co (II) centres, the green octahedral representing the Ni (II) centres, the red ball oxygen atoms and the grey ball carbon atoms, Figure S5: The crystal structure of Zn/DOBDC MOF [S3].
