# Supplementary material for: Fabrication of Monodisperse Flower-Like Coordination Polymers (CP) Microparticles by Spray Technique
Source: Nanomaterials (Basel). 2017 Aug 25;7(9):237. doi: 10.3390/nano7090237 (PMC5618348; doi:10.3390/nano7090237)
Supplement: Supplementary file 1 [file nanomaterials-07-00237-s001.pdf]

Fabrication of Monodisperse Flower-Like Coordination Polymers (CP) Microparticles by Spray Technique

Wen-Ze Li [1,*,†], Yuan Zhou [2,†], Fuchun Liu [3], Yunong Li [2], Ming-Jian Xia [2], En-Hou Han [3] , Tieqiang Wang [2], Xuemin Zhang [2], and Yu Fu [2,*]

1 Department of Applied Chemistry, Shenyang University of Chemical Technology, Shenyang 110142, China
2 College of Sciences, Northeastern University, Shenyang 110819, China; m13234040986@163.com (Y.Z.); liyunong@mail.neu.edu.cn (Y.L.); xianmingjian0927@163.com (M.-J.X.); caswtq@163.com (T.W.); zhangxuemin@mail.neu.edu.cn (X.Z.)
3 Key Laboratory of Nuclear Materials and Safety Assessment, Institute of Metal Research, Chinese Academy of Sciences, Shenyang 110016, China; fcliu@imr.ac.cn (F.L.); ehhan@imr.ac.cn (E.-H.H.)
* Correspondence: liwenze@syuct.edu.cn (W.-Z.L.); fuyu@mail.neu.edu.cn(Y.F.); Tel./Fax: +86-24-82828989 (W.-Z.L.); +86-24-83687671 (Y.F.)
† These authors contributed equally to this work.

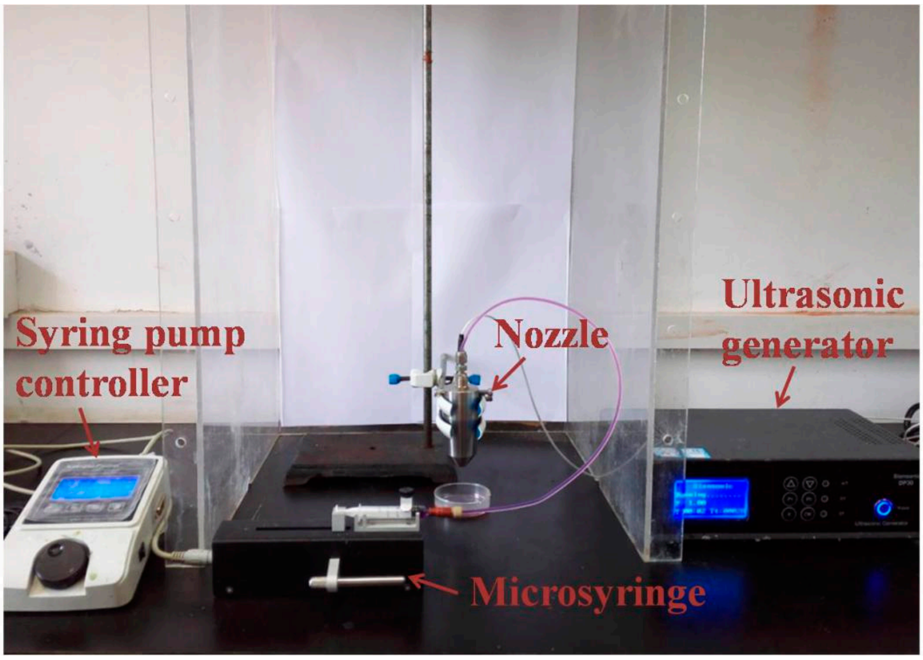

Figure S1：The picture of the fabrication equipment.

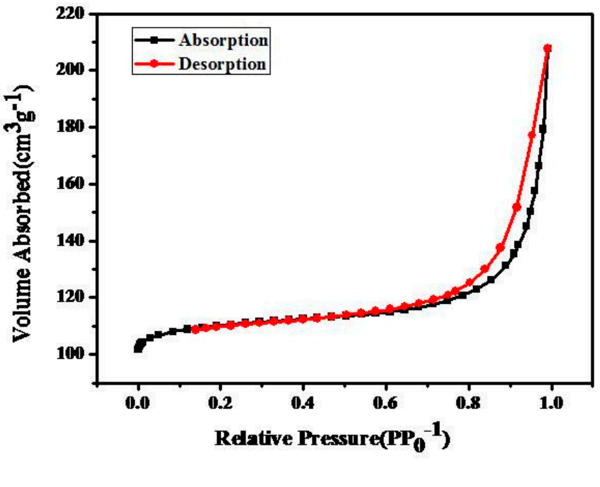

Figure S2 N_2 adsorption–desorption isotherms of the Co/BDC MOF.

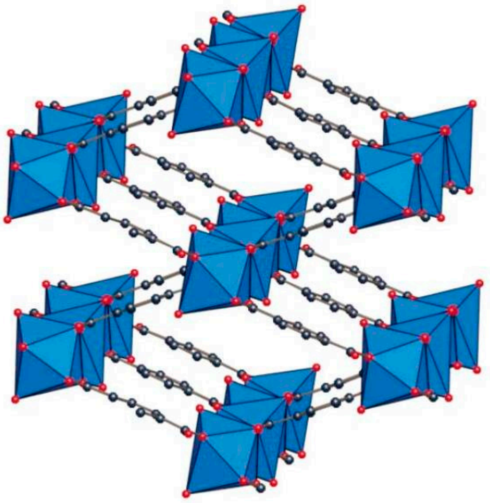

Figure S3: The crystal structure of Co/BDC MOF [S1]. Color scheme for chemical representation: blue for Co, red for O, black for C.

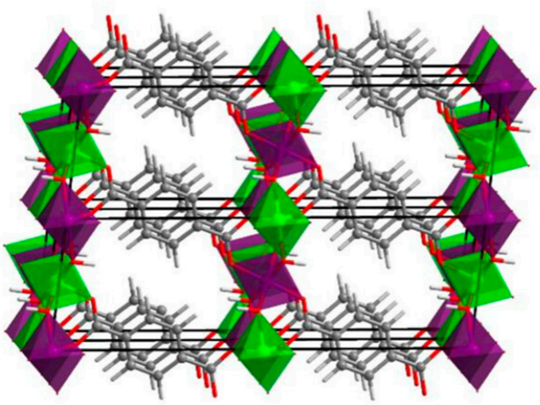

Figure S4: The crystal structure of Ni-Co/BDC MOF[S2], with the purple octahedral representing the Co(II) centres, the green octahedral representing the Ni(II) centres, the red ball oxygen atoms and the grey ball carbon atoms.

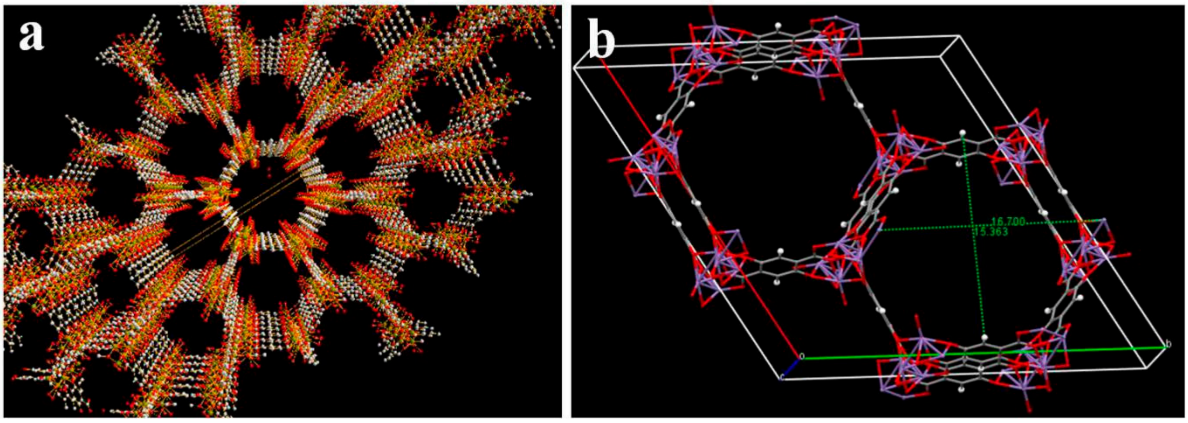

Figure S5: The crystal structure of Zn/DOBDC MOF[S3].

Supplementary References

[S1] Nathaniel, L. R.; Jaheon, K.; Mohamed, E.; Chen, B.C.; Omar,M. Y.Rod packings and metal-organic frameworks constructed from rod-shaped secondary building units. J. Am. Chem. Soc.2005, 127, 1504

[S2] Zhao, S. L.; Wang,Y.; Dong, J. C; He, C. T.; Yin, H. J.; An, P. F.; Zhao, K.; Zhang, X. F. Ultrathin metal–organic framework nanosheets for electrocatalytic oxygen evolution. Nat. Energy 2016, 1, 16184

[S3] Wong-Ng, W.; Kaduk, J. A.; Wu, H.; Suchomel, M. Synchrotron X-ray studies of metal-organic framework M2(2,5-dihydroxyterephthalate), M= (Mn, Co, Ni, Zn) (MOF74). Powder Diffr.2012, 257, 256
